# ICTV Virus Taxonomy Profile: *Thaspiviridae* 2021

**DOI:** 10.1099/jgv.0.001631

**Published:** 2021-07-30

**Authors:** Jong-Geol Kim, Khaled S. Gazi, Mart Krupovic, Sung-Keun Rhee, ICTV Report Consortium

**Affiliations:** ^1^​ Department of Biological Sciences, Wonkwang University, Iksan, 54538, Republic of Korea; ^2^​ Department of Biological Sciences and Biotechnology, Chungbuk National University, Cheongju 28644, Republic of Korea; ^3^​ Archaeal Virology Unit, Institut Pasteur, Paris 75015, France

**Keywords:** ICTV Report, taxonomy, *Thaspiviridae*

## Abstract

Members of the family *Thaspiviridae* have linear dsDNA genomes of 27 to 29 kbp and are the first viruses known to infect mesophilic ammonia-oxidizing archaea of the phylum Thaumarchaeota. The spindle-shaped virions of Nitrosopumilus spindle-shaped virus 1 possess short tails at one pole and measure 64±3 nm in diameter and 112±6 nm in length. This morphology is similar to that of members of the families *Fuselloviridae* and *Halspiviridae*. Virus replication is not lytic but leads to growth inhibition of the host. This is a summary of the International Committee on Taxonomy of Viruses (ICTV) Report on the family *Thaspiviridae,* which is available at ictv.global/report/thaspiviridae.

## Virion

**Fig. 1.**
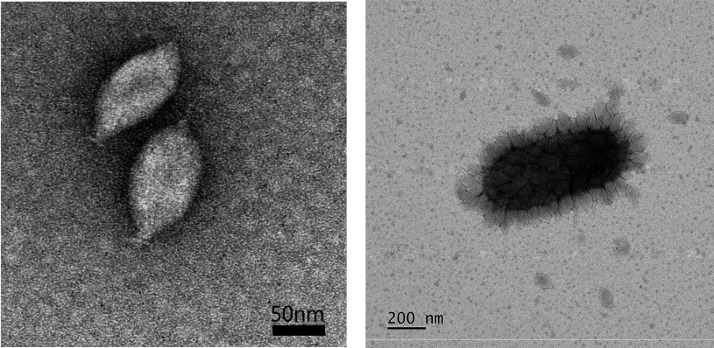
(Left) Transmission electron micrograph of negatively-stained virions of Nitrosopumilus spindle-shaped virus 1. (Right) Virus particles attached to the surface of a host cell.

The virion of Nitrosopumilus spindle-shaped virus 1 is 64±3 nm in diameter and 112±6 nm in length with a short tail at one pole (Table 1, Fig. 1). The predicted major capsid protein (81 amino acids) contains two highly hydrophobic α-helical regions. The spindle-shape morphology of the virion is very similar to those of members of the families *Fuselloviridae* [1] and *Halspiviridae* [2], which infect hyperthermophilic and hyperhalophilic archaea, respectively. When associated with the host cells, some virions are observed as elongated structures, with long thin tails connected to the cell surface [3, 4].

**Table 1.** Characteristics of members of the family *Thaspiviridae*

Example:	Nitrosopumilus spindle-shaped virus 1 (MK570053), species *Nitmarvirus NSV1*, genus *Nitmarvirus*
Virion	Spindle-shaped, measuring 64±3 nm in diameter and 112±6 nm in length, with short fibres at one pole.
Genome	Linear dsDNA (27–29 kbp) with 176 bp terminal inverted repeats
Replication	Non-lytic, chronic infection. Protein-primed family B DNA polymerase is involved in viral DNA replication
Translation	Not characterized
Host range	Ammonia-oxidizing archaea of the genus *Nitrosopumilus*
Taxonomy	Single genus with one species

## Genome

**Fig. 2.**
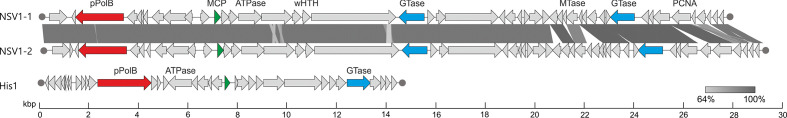
Genome maps of Nitrosopumilus spindle-shaped virus 1 isolates NSV1-1 and NSV1-2, and of the haloarchaeal halspivirus His1. Shared ORFs are connected by shaded areas based on sequence identity. Functionally equivalent (but not necessarily homologous) genes are indicated with matching colours. Filled circles indicate terminal inverted repeats. pPolB, protein-primed family B DNA polymerase; MCP, major capsid protein (putative); wHTH, winged helix-turn-helix; GTase, glycosyltransferase; MTase, DNA methyltransferase; PCNA, proliferating cell nuclear antigen.

The genome of Nitrosopumilus spindle-shaped virus 1 is linear dsDNA (27–29 kbp), terminating with 176 bp inverted repeats (Fig. 2). The virus genome is predicted to carry 48 genes. With one exception, the proteins predicted to be encoded by Nitrosopumilus spindle-shaped virus 1 are unrelated to those of other archaeal and bacterial viruses. The exception, protein-primed B DNA polymerase, is also found in several groups of archaeal viruses and non-viral mobile genetic elements which, similar to Nitrosopumilus spindle-shaped virus 1, have linear genomes with terminal inverted repeats [4].

## Replication

The virus establishes a chronic infection and its replication is not lytic, with virions being continuously extruded into the environment [4]. The virus genome is likely to be replicated by the virus-encoded protein-primed family B DNA polymerase, as has been inferred for haloarchaeal halspiviruses [2] and crenarchaeal ampullaviruses [5]. In addition, the virus encodes a proliferating cell nuclear antigen which is also likely to be involved in viral genome replication.

## Taxonomy

Current taxonomy: ictv.global/taxonomy. The single genus *Nitmarvirus* includes one species *Nitmarvirus NSV1*.

## Resources

Full ICTV Report on the family *Thaspiviridae*: ictv.global/report/thaspiviridae.
